# Are Conscientious Refusal and Conscientious Provision Mutually Exclusive? A Critique of Kelusa and Giubilini's Argument

**DOI:** 10.1111/bioe.70055

**Published:** 2025-11-18

**Authors:** Tzofit Ofengenden

**Affiliations:** ^1^ Program in Medical Ethics and Human Values and the MS Program in Bioethics and Medical Humanities at Tulane University School of Medicine New Orleans Louisianan USA

**Keywords:** conscientious objections, conscientious provision, conscientious refusal, healthcare providers, legal protection, symmetry thesis

## Abstract

This article challenges the claim that conscientious refusal and conscientious provision in healthcare are mutually exclusive and thus asymmetrical. While US law protects healthcare providers who refuse to perform medical services on moral or religious grounds, it offers no equivalent protections to those who feel morally compelled to provide care when the service is legally prohibited. This legal asymmetry has become more pressing following the Dobbs v. Jackson Women's Health Organization decision, which overturned Roe v. Wade and triggered a wave of state‐level abortion bans. Responding to recent arguments by Kulesa and Giubilini, who contend that the symmetry thesis is false and that conscientious refusal and provision cannot be justified within the same professional framework, this article argues that their position misrepresents the symmetry and constructs a strawman. It demonstrates that refusal and provision are not intended to apply simultaneously in identical cases but rather arise in distinct legal and institutional contexts. The article also critiques the rigid distinction between “pathocentric” and “interest‐centric” models of medicine, arguing that professional goals in medicine are plural, contested, and often internally conflicted. Denying moral and professional legitimacy to conscientious provision while protecting conscientious refusal undermines ethical consistency, professional integrity, and respect for moral diversity. In a society with conflicting moral frameworks, protecting both negative and positive conscience claims is necessary to uphold justice, respect provider autonomy, and ensure patient care. The symmetry thesis, understood as the equal moral and legal status of conscience‐based refusal and provision, is both defensible and essential.

In the last decade, more scholars have called for accommodating and protecting conscientious provisions. Some scholars argue that, while federal and state laws protect conscientious refusals, allowing healthcare providers to refrain from participating in legal medical services that conflict with their moral beliefs, there are no equivalent protections for providers who insist on performing procedures they consider morally necessary and that are requested by patients, but are prohibited by institutional policies or state laws. Some proponents of protecting conscientious provisions argue that since conscientious refusals are legally recognized, conscientious provisions should also be legalized, especially in the wake of the Supreme Court's ruling in Dobbs v. Jackson Women's Health Organization (2022), which overturned Roe v. Wade, eliminated the constitutional right to abortion, and prompted new state‐level abortion bans. The changing medical‐legal situation of abortion care necessitates a reevaluation of protections for healthcare providers (HCPs) who act on their conscientious provision [1]. While the original amendments protecting conscientious objections were enacted in response to the 1973 Supreme Court ruling that abortion was a constitutional right, no equivalent amendment has been enacted following the Dobbs decision to protect HCPs who feel morally obligated to provide abortions in states where it is now banned. These scholars argue that this creates an unjustified asymmetry, where the legal system accommodates conscience refusals but not conscience provisions [1, 2, 3, 4, 5, 6, 7, 8]. Kulesa and Giubilini, in their article Conscientious Refusal or Conscientious Provision: We Can't Have Both, critique the symmetry thesis, the idea that conscientious provision of illegal medical services should receive the same justification and legal protection as conscientious refusal to provide legal medical services. They argue that conscientious refusal and conscientious provision are mutually exclusive within the same case and professional model, making the symmetry thesis false [9]. In this article, I will begin by discussing the legal‐medical asymmetry regarding conscientious objections. I will then examine Kulesa and Giubilini's argument and assess the validity of their thesis. I will argue that while conscientious refusal and conscientious provision are mutually exclusive within the same case and professional model under the same legal framework, this applies only when the factual and legal circumstances are identical. However, this constitutes a strawman fallacy and fails to disprove the validity of the symmetry thesis.

## 1 What Is the Asymmetry Between Conscientious Refusal and Conscientious Provision?

In addition to federal laws protecting conscientious objectors, most states have enacted additional laws safeguarding HCPs who refuse to provide medical services that violate their religious or moral beliefs. These services often include reproductive health services such as abortion, sterilization (hysterectomy, tubal ligation, and vasectomy), birth control, and emergency contraception [10]. However, these protections extend beyond reproductive health and also cover gender‐affirming care, end‐of‐life care, stem cell research, and other medical services [11]. While state laws vary in the types and extent of protections they offer, conscience laws in thirty‐three states shield HCPs from criminal prosecution, civil liability, professional discipline, employment discrimination, and denial of public or private funding when they refuse to provide care based on conscientious objections [12, p. 1255].

For example, Mississippi's law allows healthcare providers to refuse participation in “any phase of patient medical care, treatment, or procedure, including, but not limited to, patient referral, counseling, therapy, testing, diagnosis or prognosis, research, instruction, prescribing, dispensing or administering any device, drug, or medication, surgery, or any other care or treatment rendered by healthcare providers or healthcare institutions” [13].^1^ These protections remain in place even when refusals violate the standard of care and cause significant harm to patients. This includes situations where failing to disclose a medically indicated alternative would typically violate informed consent principles [6, pp. 1049–1950, 12, p. 1280] or, in more severe cases, could constitute discrimination, negligence, malpractice, or wrongful death [12, p. 1256]. According to Sawicki's study, 7 of the 33 states that provide civil liability protection to conscientious refusers also exempt them from criminal prosecution for actions that would normally be classified as felonies, such as recklessly endangering or abandoning patients. These laws prevent patients from seeking compensation for injuries caused by a provider's conscience‐based refusal to deliver services considered standard medical care [12, pp. 1256, 1273, 1276, 1280, 1286, 1311–1312, 6, p. 1049]. Conscientious refusers are only held accountable when their inaction clearly and directly threatens a patient's life. By contrast, HCPs who feel compelled to follow their conscience and provide prohibited medical services receive no legal protection from termination, license revocation, fines, or imprisonment [14, 15]. One such case is Dr. Caitlin Bernard, an Indiana OB‐GYN, who was reprimanded and fined for performing an abortion on a 10‐year‐old Ohio girl who had been raped [16]. Another example is doctors who feel morally obligated to assist women in states where abortion is banned and risk losing their license or facing criminal charges for providing abortion pills by mail to patients.^2^ Since miscarriage treatment involves the same procedures as abortion, states that implemented abortion bans after the Dobbs decision in July 2022 permit the removal of a dead fetus or embryo but impose highly restrictive medical emergency exceptions for miscarriages when fetal cardiac activity is still detectable. These exceptions, often limited to cases where the procedure is necessary to “prevent death” or “preserve the life” of the pregnant person, create significant uncertainty for physicians, as it remains unclear how severe a patient's condition must be for the exception to apply [21, 22, 23].

As a result of these vague laws, physicians may withhold or delay miscarriage care when fetal cardiac activity is still detectable, fearing prosecution, imprisonment, monetary fines, and loss of their medical license. This delay in care can lead to life‐threatening complications, putting the health and safety of pregnant individuals at serious risk [24, 25, 26]. Thus, abortion bans force HCPs to choose between committing a crime and violating their professional duty to uphold patient autonomy, protect health, and follow their ethical conscience. The closest legal protection for conscientious provision is the abortion shield laws, enacted in 23 states and Washington, D.C.^3^ These abortion shield laws protect HCPs, including licensed doctors, nurse practitioners, and midwives, allowing them to prescribe and send abortion pills to patients in states where abortion is illegal [28, 29, 30, 31]. Conflicts between law and individual conscience also arise in end‐of‐life care. Dr. Barbara Morris, a geriatric physician, was fired by Centura Health, a Christian hospital in Colorado, for seeking to provide medical aid in dying to Neil Mahoney, a patient with advanced, incurable gastrointestinal cancer. Morris filed a court order challenging Centura's policy on the grounds that it conflicted with Colorado law, but the hospital terminated her within 5 days. Her attorney, Jason Spitalnick, clarified that Mahoney intended to die at home, not in the hospital. Nevertheless, Centura prohibits its doctors from participating in assisted dying in any form, enforcing a policy stating that Catholic healthcare providers “may never condone or participate in euthanasia or assisted suicide in any way” [32].^4^ While conscientious refusers are not forced to choose between breaking the law and protecting their patients’ interests [34, 6, p. 1034, 1, pp. 908–909] conscientious providers have no legal protection that allows them to practice medicine in accordance with their conscience. Only those who refuse to provide care are granted immunity from termination, legal claims such as malpractice and breaches of informed consent, even from criminal charges like endangering or abandoning patients, even when such refusals violate patients’ rights and cause harm [35].^5^

## 2 What Is the Symmetry?

Critics of the asymmetry thesis do not necessarily claim that conscience‐based refusals and provisions are equally justified. One can support symmetrical protections for healthcare providers even if the underlying justifications differ. In fact, most critics do not argue for perfect symmetry but rather contend that legal protections should not apply exclusively to refusals, creating an imbalance in which only refusals are protected. Fox, for example, who criticizes the asymmetry, claims that we should “level down the near‐absolute protections for conscientious refusers, while leveling up protections for conscientious providers that are virtually absent [6, p. 1039]. Some critics of the asymmetry thesis, including the author of this article, argue that conscientious provision may, in many cases, be more justified than conscientious refusal. As Fox notes, “conscientious provision honors patients' wishes, while conscientious refusal overrides them” [6, p. 1032]. In line with this reasoning, I contend that conscientious provision is often more ethically defensible because it serves the patient's needs and well‐being, whereas conscientious refusal primarily protects the moral integrity of the provider, potentially at the expense of the patient. While this asymmetry may support a stronger justification for provision, it remains important to respect the diversity of ethical perspectives in healthcare and to allow space for both forms of conscientious objection.

Ryan et al. do not advocate for symmetry either but argue that, in the wake of the Supreme Court's overturning of Roe v. Wade and ensuing abortion restrictions, conscientious providers also deserve respect. They contend that accommodations for conscience should be expanded to include not only refusals but also provisions [1]. Wicclair, for example, criticizes moral asymmetry for overlooking the moral integrity of some healthcare providers. He argues that the same reasons used to justify conscientious refusals should also apply to conscientious provisions [7, p. 16]. Similarly, Sepper contends that such imbalanced legislation is fundamentally and conceptually incoherent. In a liberal, pluralistic society, respect for an individual's moral and freedom of conscience requires treating different claims of conscience equally [3, pp. 1526, 1530]. Fritz also criticizes the asymmetry and claims that the same criteria that justify protecting conscientious refusals to provide abortion also justify protecting positive conscientious appeals regarding abortion [5, pp. 46–59]. Brummett also claims that we have reasons to protect conscientious provisions and that this asymmetry creates a seemingly unjustified double standard regarding clinicians’ conscience under the law [4, pp. 136–142]. While it is true that those who defend the permissibility of conscientious refusal may not support conscientious provisions for abortion, and vice versa. However, some scholars argue for symmetry in the sense that different moral beliefs should be tolerated. They contend that healthcare providers should not be forced to act against their core beliefs as long as patients have access to the necessary services.

For Kulesa and Giubilini, in their article, Conscientious refusal or conscientious provision: We can't have both, (2024) the symmetry thesis is that “Some authors argue that it is professionally permissible for clinicians to conscientiously provide abortion services, even if providing services would break the law, because clinicians are already allowed to conscientiously refuse to provide certain services to those who are legally eligible to receive them. Call this the professional symmetry thesis. Others have argued that, if conscientious refusal is legally protected, so should conscientious provision, at least for certain types of medical interventions despite the potential for internal contradictions in the relevant laws. Call this second claim, the legal symmetry thesis” [9, pp. 445–446].^6^ Kulesa and Giubilini present others’ calls for legal and professional symmetry as if such symmetry would “break the law” and create “internal contradictions in the relevant laws.” This framing falsely suggests that legal symmetry inherently leads to lawbreaking or instability. Yet the current asymmetry, where refusal is protected but provision is not, is itself a legal inconsistency. If clinicians may refuse care on moral grounds, even when patients are legally entitled to it, then denying protection to those who provide care on moral grounds reflects an unequal application of the law. Lawbreaking is not inherent to conscientious provision; it results from the absence of legal recognition. If provision were protected, no violation would occur, just as refusal is not considered lawbreaking when legally permitted, even though it involves the denial of lawful services. Without such protection, refusal itself would be unlawful. Both conscientious refusal and conscientious provision introduce potential legal and professional contradictions. However, such contradictions are not in themselves a reason to resist reform. Legal systems routinely navigate tensions between privacy and security, free speech and harm, and patient autonomy and provider discretion. Kulesa and Giubilini, continuing their argument by focusing solely on professional symmetry and omitting the legal dimension, argue that, based on the goals of medicine, conscientious refusal and conscientious provision are mutually exclusive, and that the professional symmetry thesis is therefore false. However, whether professional and legal symmetry can be meaningfully separated in this context is doubtful. While I maintain that such a separation is untenable and will discuss this further, a fuller examination lies beyond the scope of this article.

## 3 What Do They Mean When They Claim That Conscientious Refusal and Conscientious Provision Are Mutually Exclusive?

They argue that conscientious refusal and conscientious provision are mutually exclusive within the same case and professional model. That is these two forms of objection cannot be applied simultaneously. While they are correct in asserting that both appeals to conscience are mutually exclusive in a single case, this does not necessarily invalidate the symmetry thesis.

Kulesa and Giubilini attempt to dismantle the symmetry thesis by distinguishing between moral and professional justification. They reject the moral version, arguing that once the moral permissibility of a service (e.g., abortion) is established, refusal and provision are not equally justified. This claim is contentious, as the moral permissibility of many medical services, such as abortion, is not firmly established. Not only are there conflicting moral views, but these views can also shift with subtle contextual factors. However, since their focus is on professional justification, I will set aside the moral issue as well and concentrate on their professional argument.

Kulesa and Giubilini interpret the professional symmetry thesis to mean that conscientious refusal is professionally justified if and only if, and to the same extent as, conscientious provision. They broadly divide the goals of medicine into two categories: “pathocentric” and “interest‐centric.” By pathocentric, they mean that the goal of medicine is focused on and limited to healing—treating disease and restoring health (preventing and treating pathologies). According to them, examples of services that align with this goal include vaccines, routine check‐ups, chemotherapy, and palliative care. However, procedures such as abortion of a healthy pregnancy or medical assistance in dying do not fall within this category. In contrast, by interest‐centric, they mean that the goal of medicine is broader and focuses on enhancing the patient's well‐being, which includes respecting patient autonomy, personal goals, or quality of life—even outside disease treatment. This perspective encompasses abortion and other medical services aimed at benefiting the patient's self‐interest and overall well‐being. For instance, refusing to provide a pregnant person with an abortion to terminate a healthy pregnancy would, from an interest‐centric perspective, contradict the goals of medicine, as it would prevent the person from exercising their autonomous preferences. Kulesa and Giubilini argue that if refusal is professionally justified, then provision of the same service cannot be, since both actions cannot be grounded in the same professional goals. In their view, refusal and provision are mutually exclusive within a single case and cannot coexist within a consistent professional framework. A healthcare provider, they contend, cannot simultaneously uphold conflicting goals of medicine by engaging in both conscientious refusal and provision in the same situation. While they are right that a healthcare provider cannot simultaneously uphold conflicting goals of medicine, it remains unclear why we should not examine the symmetry thesis by considering providers with different views operating under different legal frameworks.

## 4 Misframing the Case: Why Legal Contexts Matter for Conscience‐Based Provision and Refusal

While we might be able to separate the moral and professional aspects of the debate, separating the professional model from the legal framework, as Kulesa and Giubilini do, undermines the ability to assess the validity of the symmetry thesis under varying legal circumstances. Suppose a pregnant person requests an abortion because the fetus has been diagnosed with anencephaly, a fatal neural tube defect in which the fetus lacks parts of the brain and skull. Babies with anencephaly are often stillborn or die shortly after birth. However, some states have enacted near‐total abortion bans with limited or no exceptions for severe fetal conditions.^7^ Thus, in these states, parents are forced to continue the pregnancy despite knowing their baby will not survive. If the state where the pregnant person resides allows these types of abortions, healthcare providers may refuse to perform the procedure based on their moral or religious beliefs, viewing it as killing a fetus that still has a heartbeat. However, if the pregnant person in the same situation lives in a state that bans abortion as long as the fetus has a detectable heartbeat and does not pose a risk to the pregnant person's health, a HCP may feel that denying the abortion violates their moral conscience. They may believe that respecting the pregnant person's autonomy is essential and that offering abortion is necessary; otherwise, it would be equivalent to forcing someone to continue a nonviable pregnancy, imposing significant physical, emotional, and financial burdens. This presents the same medical case under different legal frameworks, which challenges the separation between the professional and legal aspects of symmetry, since the status of professional standards is shaped and enabled by legal frameworks, leading to opposing moral responses from HCPs. One provider may believe it is wrong to terminate the life of a fetus, while another may believe it is wrong to override a pregnant person's autonomy in this situation. By setting aside the legal framework and focusing solely on the professional model, the authors obscure how the symmetry thesis functions in practice. Their claim that refusal and provision cannot both be justified under the same goal of medicine, presumably within the same legal framework, fails to account for the complexity of real‐world cases. This narrow perspective overlooks the crucial interplay between legal and professional domains that shape conscience‐based objections in healthcare. In reality, conscientious refusal and conscientious provision are not mutually exclusive when considered across different professional interpretations of medicine or under varying legal frameworks. If we interpret their definition of “the same case” as referring to an identical medical situation, under identical goals of medicine and identical legal circumstances, then their argument holds. For instance, if we consider the case of a pregnant person carrying a fetus with anencephaly in a state where abortion is completely banned, and the goals of medicine align with a pathocentric model, the only relevant appeal to conscience would be conscientious provision, as conscientious refusal would be irrelevant in this scenario. Under these exact same medical and legal conditions, conscientious refusal and conscientious provision would indeed be mutually exclusive. It would be impossible to have both appeals to conscience at the same time in the same context. However, one might question the significance of this argument. If Kulesa and Giubilini define “the same case” strictly as one occurring under identical legal circumstances and within the same model of medicine, it is unclear what their argument actually contributes.

This would amount to a strawman argument, as it does not meaningfully engage with the broader issue of how conscientious refusal and conscientious provision function under different legal frameworks. Appeals to conscience—conscientious refusals and conscientious provisions—cannot be applied to the same case simultaneously, and no one argues that they should be under identical legal circumstances or within the same professional model of health. Those advocating for symmetry have never argued for this kind of equivalence. Rather, most critics of asymmetry and proponents of accommodating conscientious provision do so because privileging some HCPs over others is unjustified, especially when the legal framework violates patients’ rights and may cause harm. Kulesa and Giubilini's argument is irrelevant, as they argue against a case that does not exist and logically cannot exist. These two forms of conscience are not intended to apply to the same case under the same legal framework, but rather under different legal frameworks. States and institutions operate with different goals of medicine, which are reflected in their legal frameworks and regulations. So, while they are correct that conscientious refusal and conscientious provision are mutually exclusive within the same case (when the legal situation is exactly the same), their claim does not challenge the symmetry thesis concerning conscientious refusal and conscientious provision. Thus, the same pregnancy situation can exist under different legal frameworks, allowing healthcare providers with differing moral beliefs to apply opposite appeals to conscience.

## 5 Is There, and Should There be, a Single Objective Goal of Medicine?

It could be that they meant to argue that if refusal is professionally justified, then provision of the same service cannot be, because both actions cannot be supported by the same professional goals. By this, they may be assuming that there can be only one consistent and objective professional goal, and that other goals cannot be justified. So either we define the goal of medicine solely in terms of healing, or solely in terms of well‐being. If we define the goal of medicine as healing, only services that fall under that definition are justified, and vice versa. However, defining the goals of medicine solely in terms of healing or well‐being is highly problematic. Healing is an ambiguous and evolving concept, and medical goals vary across contexts. For instance, should palliative care be categorized as healing or well‐being? Its aim is not to cure but to alleviate suffering and improve the quality of life for patients with chronic or terminal conditions. Depending on the type of treatment, some may classify it under the interest‐centric model rather than the pathocentric model. Vaccines are classified within the model of healing. Yet some physicians conscientiously refuse to administer the varicella vaccine because it was developed using cell lines derived from aborted fetuses. This refusal is legally protected, even though the vaccine could fit the definition of healing. This suggests that conscientious refusal is not always tied to the goal of healing, or that the ethical landscape is more complex. Some institutions and healthcare providers oppose abortion even in cases involving pregnancy pathologies, such as severe fetal anomalies or nonviable pregnancies. Another example is pregnancy exclusions in advance directives. More than thirty states limit the enforcement of such directives if the patient is pregnant. In nine of those states, a pregnant person can be kept artificially alive against their expressed wishes, even if the fetus is not viable. Framing the ethics of care as a choice between two opposing models oversimplifies reality. Many healthcare providers do not view their work through such rigid categories. A provider may support abortion in some cases while rejecting it in others based on nuanced reasoning. The case of Sister Margaret McBride, a senior administrator and ethics committee member at St. Joseph's Hospital in Phoenix, highlights the complexity of such decisions. She was excommunicated after approving a first‐trimester induced abortion for a patient with life‐threatening pulmonary [38]. The hospital prohibited abortion even in life‐threatening cases. Labeling abortion as outside the scope of medicine may reflect cultural or religious beliefs rather than clinical reasoning that accounts for the complexity of patient needs and specific medical contexts. Such generalizations risk excluding essential aspects of medical judgment and ethical decision‐making. This form of biological reductionism ignores broader definitions of health, such as that of the World Health Organization, which includes mental and social well‐being. Forced pregnancy can seriously harm a person's psychological, emotional, and social health. Healthcare is not defined by biology alone; it involves balancing principles such as beneficence, non‐maleficence, autonomy, and justice. These values often come into tension. Kulesa and Giubilini assume an objective professional goal of medicine, but clinicians reasonably disagree about how to interpret and prioritize these values. Since the goals of medicine are plural and contested, they cannot provide a definitive basis for judging conscientious refusals.

## 6 The Professional Model of Medicine: Who Has the Authority?

The authors argue that a course of action is professionally justified if it is supported by compelling reasons grounded in the appropriate goals of medicine, whether according to the pathocentric or interest‐centric model. On this view, the model determines which actions are justified and which are not, leading them to reject the professional symmetry thesis. But beyond the earlier critique that the goals of medicine are not clearly defined, this raises an additional question: who has the authority to define those goals, the state, institutions, individual healthcare professionals, or patients? If institutions hold exclusive authority, then conscience‐based objection, whether refusal or provision, is never justified, since any deviation would undermine the official professional goal of medicine they operate under. In that case, the symmetry thesis becomes irrelevant, and no conscience‐based objection is defensible at all, since professional standards are fixed and singular. Only one correct model would exist, and thus neither form of conscientious objection could be considered legitimate. It does not seem that the authors intended this conclusion. Yet under this logic, it follows. On the other hand, if individual HCPs are permitted to follow their own professional models, then their interpretation of professional obligations overrides institutional or legal norms. But this creates a tension: if a clinician can refuse to provide care based on their interpretation of “true” medical practice, then the same reasoning must apply to those whose professional judgment compels them to provide care that the institution or state prohibits. In both cases, the healthcare provider claims their professional standard as morally authoritative. This undercuts the authors’ claim and demonstrates that the symmetry thesis is not false. Without addressing how professional standards are determined and who has the legitimate authority to define them, the authors’ position lacks a coherent foundation. It remains unclear whether individual practitioners should have the right to define standards that contradict institutional or legal frameworks. If we regard HCPs as moral agents, then we must extend protections equally to those who refuse to act and to those whose conscience compels them to act and this leads to the last argument that the symmetry thesis affirms this moral consistency in a pluralistic society.

## 7 Moral Integrity in a Pluralistic Society: Defending the Symmetry of Conscience

The authors reject moral relativism, arguing that if it were true, abortion could be both morally permissible and impermissible depending on one's standpoint, collapsing moral justification into mere personal belief. To avoid this conclusion, they assert that moral claims must be grounded in an objective standard of health rather than individual conscience. But again, if such a single, objective professional standard exists, then by the same logic, there would be no room for conscientious objection at all. While that may be the preferred model, there is no single, objective professional framework in medicine. Rather, multiple conceptions of its goals coexist, opening the door to a normative recognition of moral pluralism—or at least to the view that identifying a single objective is difficult, especially when complex circumstances shape both moral and professional judgment. If competing professional and moral frameworks exist, then both the view that abortion is morally impermissible and the view that it is morally permissible can be justified, depending on the framework adopted. As a result, both conscientious refusal and conscientious provision could be morally valid. This is not radical relativism, but it does affirm a form of moral pluralism, perhaps cultural relativism. Medicine involves weighing conflicting values such as autonomy, beneficence, non‐maleficence, and justice. As the authors themselves note, abortion debates cannot be reduced to a single moral concern, such as the status of fetal life. Other factors, like the pregnant person's bodily autonomy, must also be considered.

In addition, the assumption that “the goals of the profession” could provide a singular and authoritative standard is philosophically contested. While some scholars argue for an essential goal such as healing or beneficence, others highlight that the goals of medicine are changing and evolving and often context specific.^8^ This variability reflects the moral and cultural pluralism that shapes medical practice across institutions and societies. Relying on a single abstract goal as the exclusive basis for professional justification risks ignoring the internal diversity and interpretive flexibility present in real clinical settings. In this light, both conscientious refusal and conscientious provision can be seen as legitimate expressions of moral agency responding to complex clinical realities, each invoking different yet deeply held ethical commitments within the broader field of medicine. If we defend one as morally justified while rejecting the other, we undermine the principle of conscience protections, which are meant to safeguard moral disagreement, not enforce ideological consensus.

Their argument also overlooks the moral complexity of societies like the United States, where there is no consensus on issues such as abortion, end‐of‐life care, or reproductive services. Conscientious objection in healthcare reflects these deep moral and professional disagreements. The need to accommodate both forms of conscience arises precisely because we live in a society that is not unified in its moral beliefs. Lawmakers, healthcare professionals, and patients often hold conflicting values, sometimes at odds with institutions, legal regimes, or each other.

Respecting moral integrity in this context requires accommodating a range of religious and ethical perspectives. The original motivation behind the 1973 Church Amendment was to protect healthcare providers’ moral integrity after abortion became a constitutional right [41]. It allowed clinicians to opt out of abortion and sterilization procedures without risking their employment or licenses. The same arguments used to justify conscientious refusal, such as moral integrity, respect for deeply held beliefs, and cultural pluralism, should apply equally to conscientious provision. If a physician can refuse care based on moral conviction, another should be able to provide care on the same grounds, especially when doing so aligns with the patient's values and needs [5, p. 48, 7]. Moral integrity can be compromised both by being forced to act against one's conscience and by being prevented from acting on it when compelled by moral duty [42, 43, 44, 45]. In this context, symmetry does not mean treating the same case in contradictory ways, but recognizing that the moral integrity of all physicians deserves equal respect, not only those whose beliefs align with specific moral or religious traditions [46, 47, 6, p. 1043]. If we accept that physicians should not be emotionally or morally detached from their professional duties, this principle must apply universally, across all moral perspectives. Both conscientious refusal and conscientious provision should be accorded the same moral status. In this sense, the symmetry thesis holds and cannot be dismissed as false.

The authors are correct that conscientious refusal and provision cannot apply simultaneously within the same case, when the legal and professional contexts are identical. In states where abortion is banned, conscientious refusal becomes irrelevant because the service is already illegal, there is nothing to refuse. Instead, conscientious provision becomes morally relevant. Conversely, in states where abortion is legal, conscientious refusal becomes the operative form of moral expression. This variation does not undermine the legitimacy of accommodating both types of conscience claims. Rather, it reflects the need to protect both forms of conscience in different legal and institutional contexts.

## Conflicts of Interest

The author declares no conflicts of interest.

## Endnotes

1 See also Louisianan law, “No person shall be held civilly or criminally liable, discriminated against, dismissed, demoted, or in any way prejudiced or damaged for declining to participate in any health care service that violates his conscience.” LA Rev Stat § 40:1061.20 (2018).

2 For example, Louisiana issued arrest warrant for Dr. Margaret Carpenter from New York for prescribing abortion pills [17]. See also C. Kitchener [18], S. Taladrid [19], and Wadhwani, A [20].

3 The specific protections and provisions of these shield laws vary by state, but they generally aim to safeguard providers against out‐of‐state legal actions [27].

4 See also [33].

5 See also [36, 12, pp. 1257–1260, 6, p. 1035].

6 Kulesa and Giubilini present a simplified version of the symmetry thesis. While this is an important issue, I will not elaborate on it here due to space constraints, as my focus is on refuting their main claim that the symmetry thesis is false.

7 Texas, Alabama, Mississippi, Arkansas, Oklahoma, Louisiana, Idaho, and Tennessee. See, for example [37].

8 For an essentialist conception of the internal morality of medicine (IMM), see E. Pellegrino [39]. For a critique of IMM, see, for example, F. G. Miller and H. Brody [40].
